# Biocompatible orthodontic cement with antibacterial capability and protein repellency

**DOI:** 10.1186/s12903-021-01779-7

**Published:** 2021-08-20

**Authors:** Miao Chen, Jianru Yi, Zhihe Zhao

**Affiliations:** State Key Laboratory of Oral Diseases, National Clinical Research Center for Oral Diseases, Department of Orthodontics, West China Hospital of Stomatology, Sichuan University, #14, 3rd Section, South Renmin Road, Chengdu, 610041 People’s Republic of China

**Keywords:** Orthodontic cement, Biocompatibility, Protein repellency, Biofilms

## Abstract

**Background:**

White spot lesions (WSLs) often occur in orthodontic treatments. The objectives of this study were to develop a novel orthodontic cement using particles of nano silver (NAg), N-acetylcysteine (NAC) and 2-methacryloyloxyethyl phosphorylcholine (MPC), and to investigate the effects on bonding strength, biofilms and biocompatibility.

**Methods:**

A commercial resin-modified glass ionomer cement (RMGIC) was modified by adding NAg, NAC and MPC. The unmodified RMGIC served as the control. Enamel bond strength and cytotoxicity of the cements were investigated. The protein repellent behavior of cements was also evaluated. The metabolic assay, lactic acid production assay and colony-forming unit assay of biofilms were used to determine the antibacterial capability of cements.

**Results:**

The new bioactive cement with NAg, NAC and MPC had clinically acceptable bond strength and biocompatibility. Compared to commercial control, the new cement suppressed metabolic activity and lactic acid production of biofilms by 59.03% and 70.02% respectively (*p* < 0.05), reduced biofilm CFU by 2 logs (*p* < 0.05) and reduced protein adsorption by 76.87% (*p* < 0.05).

**Conclusions:**

The new cement with NAg, NAC and MPC had strong antibacterial capability, protein-repellent ability and acceptable biocompatibility. The new cement is promising to protect enamel from demineralization during orthodontic treatments.

## Background

Although there has been great advancement in orthodontic treatment due to the innovations in materials and techniques, practitioners still frequently face two iatrogenic problems: external root resorptions and white spot lesions (WSLs) [1]. Compared to the root resorptions that are usually less than 2 mm and therefore having no obvious effects on loss of tooth stability [2, 3], WSLs could be esthetically unacceptable to both patients and orthodontists [4]. WSLs are the manifestation of enamel demineralization caused by organic acids from biofilms aggregated around orthodontic appliances. Numerous interventions including reminder therapy, chlorhexidine rinses, diet modification, calcium-containing products and fluoride-containing products were introduced to prevent the development of WSLs. However, the effectiveness of these methods requires long-term patient compliance and has been found to be unsatisfactory [5–7]. Therefore, the approaches that are independent of patient compliance are needed to combat WSLs.

Anti-caries orthodontic cement is a promising measure to combat WSLs due to its independence of patient compliance and its proximity to biofilms. The development of anti-caries cement should counter the etiology of WSL. The formation of orthodontically induced WSLs is well documented. Briefly, salivary proteins could be adsorbed onto enamel surface adjacent to brackets and form the acquired salivary pellicle, which serves as the substratum for the attachment of oral bacteria and is critical for biofilm formation [8]. The mature biofilm consumes carbohydrates and produces organic acids, which further result in enamel demineralization manifesting as WSLs [9]. Therefore, an ideal orthodontic cement to combat WSLs should have protein-repellent property to decrease the salivary protein adsorption and antibacterial ability to inhibit the acid production.

The nano-scale materials have been found effective to improve the performance of dental materials [10, 11]. Nano silver (NAg) possesses a long-lasting antibacterial property [12]. The addition of NAg renders strong antibacterial capability to dental composite and root canal sealer [13]. A concern of using NAg in dental material is the biocompatibility [14]. Recently, we incorporated N-acetylcysteine (NAC) into a NAg-containing orthodontic cement and obtained favorable biocompatibility [15]. As an antioxidant, NAC alleviates the cumulative effects of oxidative stress on cells, thus decreasing the cytotoxicity induced by NAg [15]. The combined use of NAg and NAC provides a safe approach to achieve antibacterial activity in orthodontic cement [15].

Another approach to combat WLSs is to develop protein-repellent cement. The rough surface of cement results in more bacteria accumulation. The acquired salivary pellicle that is produced by salivary protein adsorption acts as the substratum of the bacteria attachment in oral cavity [16]. It would be beneficial to develop a cement that repels protein adsorption, thus inhibiting bacterial adhesion. 2-metha-cryloyloxyethyl phosphorylcholine (MPC) has a phospholipid polar group in the side chain thereby possessing the ability to repel protein adsorption [17]. MPC has been recently incorporated into several dental materials and was found to be effective to repel protein [18, 19].

Accordingly, the objective of the present study was to use NAg, NAC and MPC to formulate a novel orthodontic cement with acceptable bonding strength, antibacterial and protein-repellent capabilities, and favorable biocompatibility. It was hypothesized that: (1) Enamel bond strength of novel orthodontic cement containing NAg, NAC and MPC would be within the acceptable range as recommended in the literature; (2) The new orthodontic cement substantially decrease biofilm growth and acid production, compared to commercial control; (3) The new cement would decrease the protein adsorption, compared to commercial control; (4) The new cement would have acceptable biocompatibility.

## Methods

### Ethical approval

The study protocol was approved by the Institutional Review Board of the West China Hospital of Stomatology. Written informed consent was obtained from each tooth donor. The research was performed in accordance with the Declaration of Helsinki.

### Synthesis of NAg

**Fig. 1:**
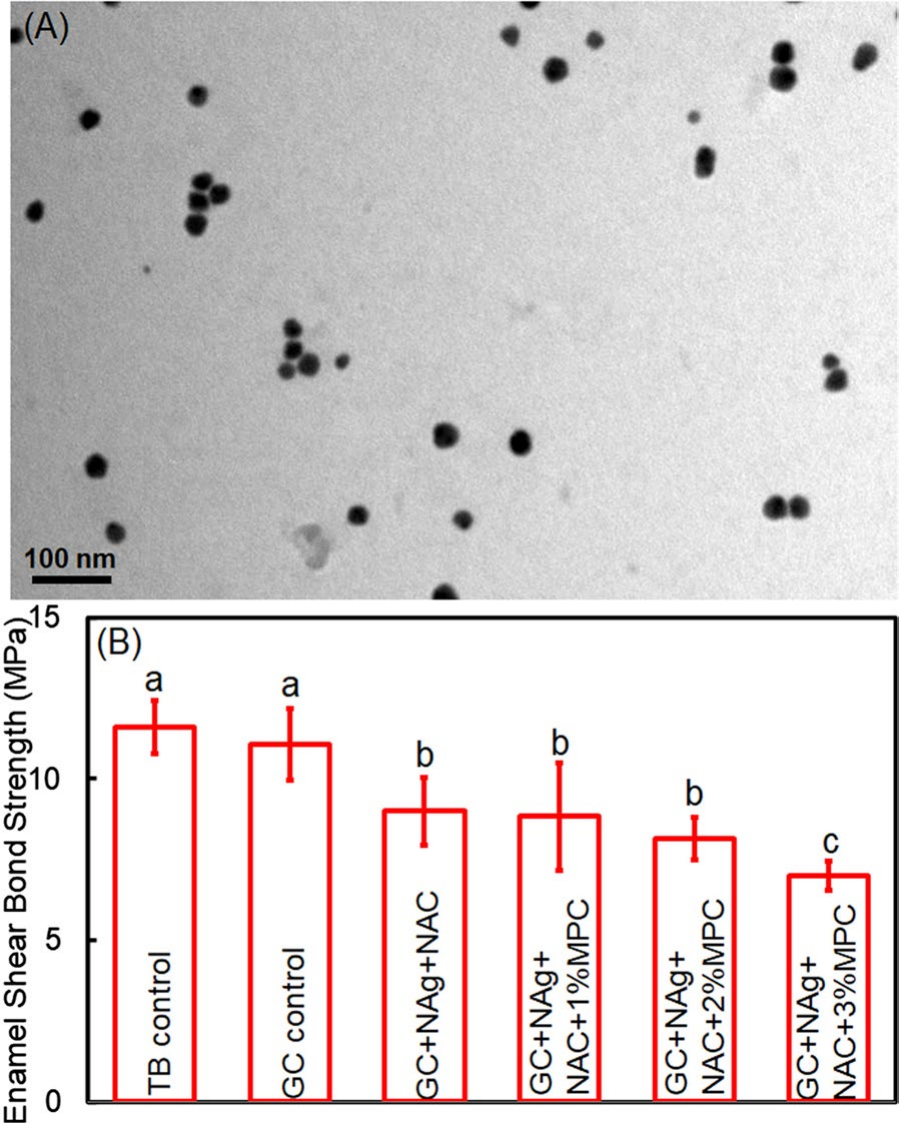
NAg particles and enamel shear bond strengths. **A** Typical TEM image of NAg particles. **B** Enamel shear bond strengths of cements. The incorporation of MPC had no adverse effects on bond strength until the mass fraction reached 3% (*p* < 0.05). The same letters indicate no statistical difference between the groups (*p* > 0.1), while the different letters indicate a significant difference (*p* < 0.05)

The NAg was commercially obtained (Shanghai So-Fe Biomedicine Co., Ltd, China. The NAg was synthesized using a conventional sodium citrate reduction method according to the manufacturer’s instructions. A transmission electron microscopy (TEM; JEOL Ltd, Tokyo, Japan) was used to characterize the morphology and size of the nanoparticles. According to the TEM observation, the average diameter of NAg was approximately 20 nm (Fig. 1A).

### Preparation of cement containing NAg, NAC and MPC

A resin-modified glass ionomer cement (GC Ortho LC, Fuji, Aichi-ken, Japan) was used as the parental system and denoted as GC. NAg was added into GC at a mass faction of 0.15%. NAC (Sigma-Aldrich, St Lousi, Mo, USA) was added into NAC at a mass faction of 20%. The mass fraction of NAg and NAC were based on our previous study showing favorable bonding strength of the orthodontic cement [15].

MPC was commercially obtained (Sigma-Aldrich), and was incorporated into the cement containing NAg and NAC at a mass fraction of 1%, 2% and 3%. The mass fractions higher than 3% were not used to avoid compromising the enamel bond strength.

### Enamel shear bond strength (SBS) testing

Thirty-six non-carious human premolars extracted for orthodontic reasons were collected and randomized to six groups for enamel SBS testing. Each premolar was vertically embedded into self-curing acrylic resin (Fuji). The buccal surface of each premolar was etched using 37% phosphoric acid gel (XihuBiom, Hangzhou, China) for 30 s, and then thoroughly dried by an air stream. A commercial cement (Transbond XT, 3 M unitek, Monrovia, CA, USA) was used as the control. Each cement was used to bond a metal premolar bracket (Shinye, Hangzhou, China) to the center of buccal surfaces. The following groups were recruited in the SBS testing:

1. Transbond XT control (referred to as TB control)
2. GC control
3. GC + 0.15% NAg + 20% NAC (GC + NAg + NAC)
4. GC + 0.15% NAg + 20% NAC + 1% MPC (GC + NAg + NAC + 1%MPC)
5. GC + 0.15% NAg + 20% NAC + 2% MPC (GC + NAg + NAC + 2%MPC)
6. GC + 0.15% NAg + 20% NAC + 3% MPC (GC + NAg + NAC + 3%MPC)

For each SBS testing, a chisel that was connected to a universal testing machine was positioned right above the bracket base to apply a load to bracket base until the bracket was detached. The SBS was determined by dividing the peak load by the bracket contact surface area with enamel.

### Specimen preparation

The SBS test showed that the bond strength decreased to a clinically unacceptable level when the mass fraction of MPC exceeded 2%. Hence, the following four groups were prepared for the subsequent experiments: (1) TB control; (2) GC control; (3) GC + NAg + NAC; (4) GC + NAg + NAC + 2%MPC.

The cement pastes of the four groups were placed in each well of a 96-well plate cover and light cured for 1 min. The cement disks were approximately 8 mm in diameter and 0.6 mm in thickness. The cement disks were immersed in distilled water and stirred using a magnetic bar at a speed of 100 rpm for 1 h to release the uncured monomers [20].

### Measurement of protein adsorption on cements

A micro bicinchoninic acid (BCA) method was adopted to measure protein adsorption. Briefly, the disks were immersed in bovine serum albumin (BSA; Sigma-Aldrich) solution with a concentration of 4.5 g/L at 37 ℃ for 2 h. The BSA adsorbed on disk surface were detached by sonication and analyzed using a protein analysis kit (micro BCA, Fisher Scientific, Pittsburgh, PA, USA) spectrophotometrically.

### Biofilm formation

*S mutans* (ATCC 700610) were incubated in brain heart infusion (BHI) broth (BD, Franklin Lakes, NJ, USA) at 37 ℃ with 5% CO_2_ aerobically overnight. The inoculum was prepared by diluting the *S mutans* suspension to approximately 10^7^ colony-forming units (CFU)/mL. The cement disks were placed into 24-well plate. 1.5 mL of inoculation was added into each well to immerse cement disk. After a 24-h culture at 37 ℃ with 5% CO_2_, the cement disks were transferred to a new 24-well plate filled with 1.5 mL of fresh BHI and incubated for another 24 h. A total of 48 h of incubation was able to form a mature biofilm on cement disks [21].

### MTT metabolic assay of biofilms

MTT assay is a method to evaluate cell survival and growth through detecting the enzymatic reduction of a yellow tetrazolium salt. The metabolic activity of biofilms on cement disks was determined by MTT assay. The cement disks with 2-day biofilms were transferred into new 24-well plates and immersed by 1 mL of MTT solution. After incubation at 37 ℃ for 1 h, the disks were transferred to new plates filled with 1 mL of dimethysulfoxide. After incubation in dark for 20 min, and absorbance of dimethysulfoxide solution at OD_540_ nm was evaluated.

### Lactic acid production of biofilms

The cement disks with 2-day biofilms were transferred to a new 24-well plates filled with 1.5 mL of buffered peptone water plus 0.2% sucrose. The specimens were incubated at 37 ℃ with 5% CO_2_ for 3 h to allow the biofilms to produce acid. The buffered peptone water was then collected to analyze the lactate content at OD_340_ spectrophotometrically. Lactic acid standard solutions were used to establish the standard curve for the conversion of OD readings.

### CFU counts of biofilms

The cement disks with 2-day biofilms were transferred into tubes filled with 2 mL of cysteine peptone water. The biofilms were collected by sonication and vortexing. The solution containing biofilms were serially diluted and then spread onto BHI agar plates. After a 48-h incubation, the number of colonies was counted.

### Cytotoxicity analysis

The cement disks were placed in 48-well plates filled with 500 μL of Dulbecco’s Modified Eagle Medium. This yielded a ratio of cement surface area to solution to be about 1.8 cm^2^/mL, which is within the range recommended by the International Standards Organization (ISO). The 48-well plates with disks were incubated at 37 ℃. The DMEM was replaced on a daily basis. The extracts of day 1, 4, and 7 were collected and stored at − 20 °C for cytotoxicity analysis.

Human gingival fibroblasts (HGFs) were used to evaluate the cytotoxicity of orthodontic cement. The HGFs were seeded into 96-well plates and cultured using the extracts plus 100 IU/mL penicillin, 100 IU/mL streptomycin and 10% fetal calf serum at 37 °C for 24 h. The HGFs incubated with the medium without extracts from cement served as the control. A MTT assay was performed to evaluate the cell density at OD_492_ nm. The OD ratios of experimental groups to control group were determined as the cell viabilities.

### Statistical analysis

SPSS 19.0 for Windows (SPSS Inc, Chicago, IL, USA) was used for statistical analysis. All data were expressed as mean ± standard deviation (sd). The normal distribution assumption of all data was identified using Shapiro–Wilk Analysis. One-way analyses of variance and Fisher’s Least Difference post-hoc test were adopted to investigate the difference among five groups. The significance level of *p* was set at 0.05.

## Results

The enamel SBS results are displayed in Fig. 1B. The SBS of TB control and GC control were higher than that of the cement containing NAg and NAC (*p* < 0.05). The SBS of the cement containing NAg and NAC was not compromised until the mass fraction of MPC reached 3% (*p* < 0.05).

**Fig. 2:**
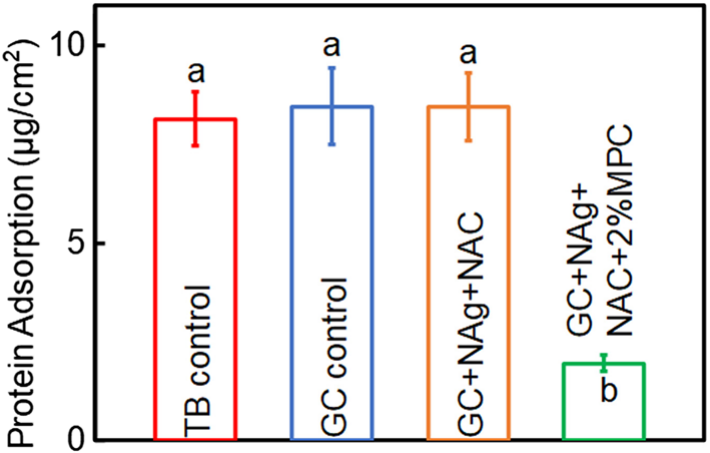
Protein adsorption on cement disks. The cements containing MPC had protein adsorption that was about 1/5 of those without MPC (*p* < 0.05). Different letters indicate a significant difference from each other (*p* < 0.05)

The protein adsorption on cement disks are plotted in Fig. 2. The group containing MPC had significantly lower protein adsorption (*p* < 0.05), which were approximately 1/5 of those three groups without MPC.

**Fig. 3:**
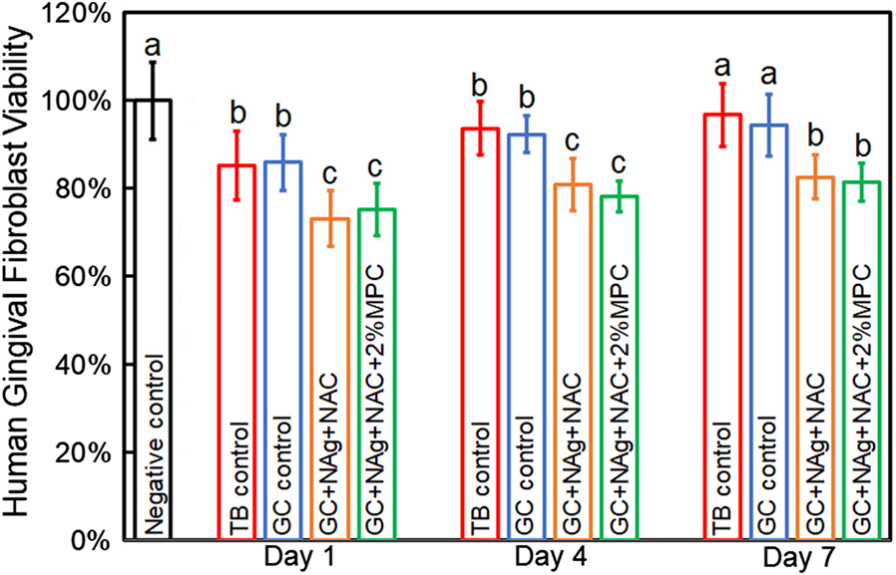
Cytotoxicity of cement disks. The incorporation of NAg and NAC yielded cell viabilities above 70% at all three time points. The addition of MPC showed no adverse effects on the cell viabilities (*p* > 0.1). Different letters indicate a significant difference from each other (*p* < 0.05)

The viabilities of HGFs against orthodontic cements are displayed in Fig. 3. No obvious differences in cell viabilities were detected between the extracts of TB control and GC control (*p* > 0.1). The addition of MPC into the cement containing NAg and NAC had no adverse effects on the cell viabilities (*p* > 0.1), yielding a cell viability of 75.1%, 78.0% and 81.3% for day 1,4 and 7 respectively, all of which were higher than the requirement of ISO (70%).

**Fig. 4:**
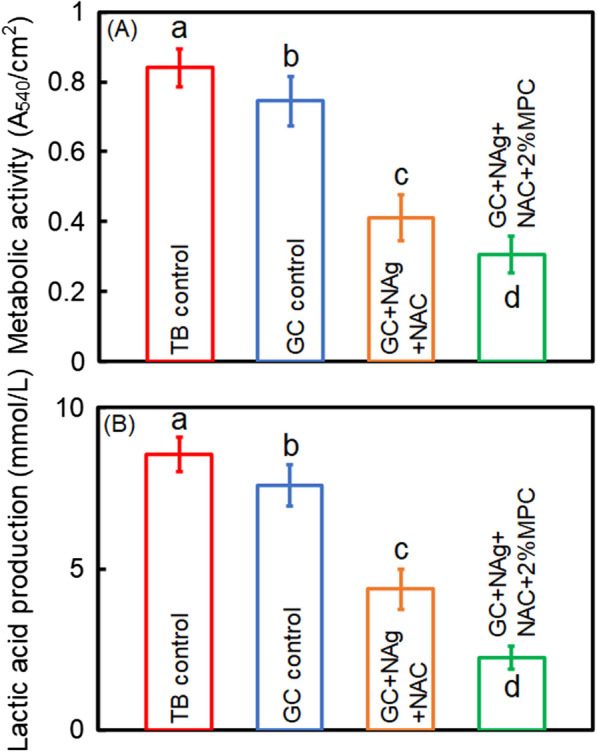
Quantitative biofilm properties on cement disks. (A) MTT assay and (B) lactic acid production. Different letters indicate a significant difference from each other (*p* < 0.05)

Figure 4 exhibits the metabolic activity and lactic acid production of 2-day biofilms on cement disks. Compared to TB control and GC control, the metabolic activity of biofilms was suppressed by NAg and NAC (*p* < 0.05), and was further suppressed in groups of NAg + NAC + 2%MPC (*p* < 0.05). Similar trends were observed in the production of lactic acid.

**Fig. 5:**
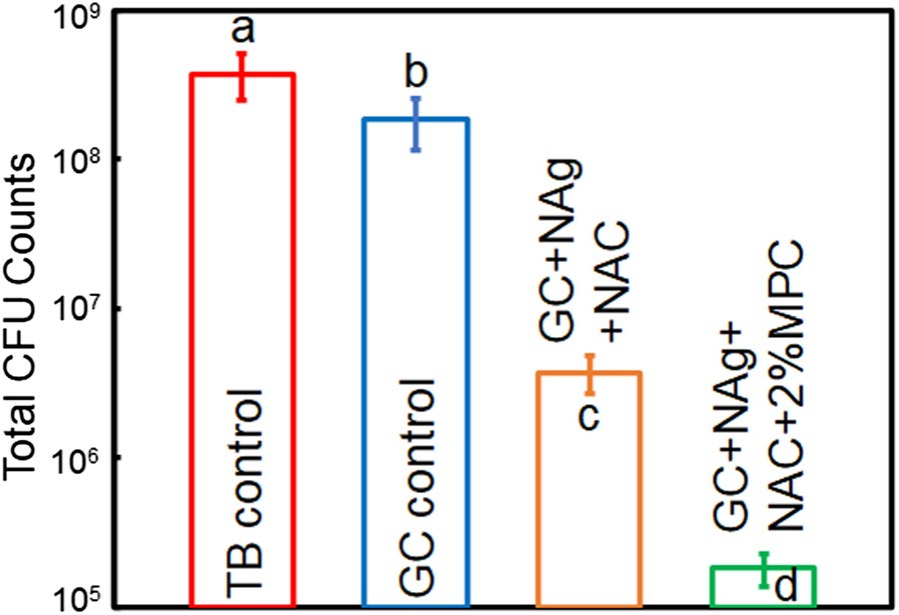
CFU counts of total *S. mutans* on cement disks. The incorporation of NAg and NAC greatly reduced CFU counts (*p* < 0.05), which was further decreased by MPC (*p* < 0.05). Different letters indicate a significant difference from each other (*p* < 0.05)

The CFU counts of 2-day biofilms are shown in Fig. 5. The CFU counts of GC control was slightly lower than that of TB control (*p* < 0.05). The addition of NAg and NAC reduced the CFU counts by approximately 2 orders of magnitude (*p* < 0.05), which was further reduced by the use of MPC (*p* < 0.05).

## Discussion

In the present study, we developed a novel nanostructured orthodontic cement containing NAg, NAC and MPC, and investigated its bonding strength, antibacterial capability, protein-repellent property and biocompatibility for the first time. The hypotheses were proven that incorporating NAg, NAC and MPC at appropriate mass fractions yielded an SBS meeting the suggested requirement for clinical application; the cement containing NAg, NAC and MPC inhibited biofilm growth and lactic acid production; the new cement greatly reduced the protein adsorption and had acceptable biocompatibility.

Efforts have been made to render orthodontic cement a strong bacterial capability in past decades. NAg and NAC were used to obtain antibacterial property in this study. The silver ion can result in bacteria death via inactivating the vital enzymes of oral bacteria and disturbing DNA replication [22]. NAC inhibits the metabolism of biofilms through suppressing cysteine utilization and disturbing intracellular redox equilibrium [23]. The incorporation of NAg and NAC reduced the metabolic activity, lactic acid production, and CFU counts by 44.8%, 42.3%, and nearly two orders of magnitude, respectively, suggesting the combined use of NAg and NAC is effective to inhibit the metabolisms of *S mutans*.

The MPC molecule has a phospholipid polar group in the side chain, thus making the MPC polymer highly hydrophilic [24]. This was suggested to reduce protein adsorption and bacterial attachment [25]. The adsorption of salivary proteins on orthodontic cement and enamel is the prerequisite of biofilm formation. The protein repellency would be beneficial for orthodontic cement to combat WSLs. Indeed, the incorporation of MPC greatly decreased the protein adsorption on cement disks, which further reduced CFU counts and lactic acid production in this study. This finding indicates the high efficiency of the combined use of NAg, NAC and MPC to suppress the development of WSLs.

An appropriate SBS is essential for a qualified orthodontic cement. The bonding strength should not be too high to hinder the removal of bracket after treatment, and not too low to keep the stability of bracket during treatment. The SBS recommended for clinical application is around 7.8 MPa [26]. In this study, the bonding strength decreased to 6.97 MPa when the mass fraction of MPC reached 3%, thus 2% MPC was used to develop the cement, yielding a bond strength of 8.13 MPa. This suggests this novel cement seems acceptable for clinical practice. Future studies are needed to determine its bonding behaviors in oral conditions.

A major concern for adding bioactive agents into dental materials is the biocompatibility. Indeed, the incorporation of NAg greatly reduced the cell viabilities in our previous study [15]. The disturbance of intracellular redox equilibrium is suggested to be a main cause of dental materials-induced cell damages [27]. The leachable monomers and agents from dental materials promotes the formation of reactive oxygen species (ROS), which causes cell death through producing acute injuries to cellular proteins and DNA [27, 28]. NAC is an antioxidant that facilitates the detoxification of ROS [29]. In this study, the combined use of NAC and NAg resulted in a clinically acceptable biocompatibility according to the ISO [30], which was not compromised by the incorporation of MPC. This finding is consistent with current evidences suggesting the favorable biocompatibility of MPC [31], and demonstrates that the combined use of NAC, NAg and MPC can be a safe approach to combat WSLs in clinical practice.

The main limitation of this study is that the in vitro condition we used can not precisely simulate the in vivo environment including the mastication and flow of saliva, which could have profound influences on the metabolism of biofilms. Hence, future in vivo studies are needed to explore the actual effects of this new cement on the development of WSLs during orthodontic treatment.

## Conclusions

The present study developed a nanostructured orthodontic cement combining the NAg, NAC nad MPC to inhibit enamel white spot lesions for the first time. The new cement had clinically acceptable enamel SBS and biocompatibility. The new cement achieved great reduction in the growth and lactic acid production of biofilms, and decreased the biofilm CFU by more than 2 logs, compared to control. Therefore, the combined use of NAg, NAC and MPC is promising to protect enamel from demineralization during orthodontic treatment, and may provide a new strategy to combat caries in other dental applications.

## Data Availability

The data of this study are available from corresponding author on reasonable request.

## Abbreviations

WSLs: White spot lesions
NAg: Nano silver
NAC: N-acetylcysteine
MPC: 2-Metha-cryloyloxyethyl phosphorylcholine
SBS: Shear bond strength
ROS: Reactive oxygen species
